# Serum alanine aminotransferase as an early marker of outcomes in patients receiving anti-PD-1 or anti-CTLA-4 antibody

**DOI:** 10.1038/s41598-021-88744-0

**Published:** 2021-05-13

**Authors:** Takeshi Azuma, Takumi Takeuchi, Yukihide Matayoshi, Shin Namiki, Tetsuya Obara, Kazuhiro Imamura, Mikio Takamori

**Affiliations:** 1 Division of Urology, Tokyo Metropolitan Tama Medical Center, 2-8-29 Musashinodai, Fuchu, Tokyo 183-8524 Japan; 2 Division of Urology, Kanto Rosai Hospital, 1-1 Kizukisumiyoshi-cho, Nakahara-ku, Kawasaki-City, Kanagawa 211-8510 Japan; 3 Division of Urology, Tomishiro Central Hospital, 25 Ueda, Tomigusuku, Okinawa 901-0243 Japan; 4 Division of Gastroenterology and Hepatology, Tokyo Metropolitan Tama Medical Center, 2-8-29 Musashinodai, Fuchu, Tokyo 183-8524 Japan; 5 Division of Thoracic Surgery, Tokyo Metropolitan Tama Medical Center, 2-8-29 Musashinodai, Fuchu, Tokyo 183-8524 Japan; 6 Division of Surgery, Tokyo Metropolitan Tama Medical Center, 2-8-29 Musashinodai, Fuchu, Tokyo 183-8524 Japan; 7 Division of Respiratory Medicine and Medical Oncology, Tokyo Metropolitan Tama Medical Center, 2-8-29 Musashinodai, Fuchu, Tokyo 183-8524 Japan; 8 Department of Urology, Tokyo Metropolitan Tama Medical Center, 2-9-2 Musashidai, Fuchu, Tokyo 183-8524 Japan

**Keywords:** Cancer immunotherapy, Gastrointestinal cancer, Lung cancer, Tumour immunology, Urological cancer

## Abstract

Immune-oncology (IO) drug therapy is effective against various types of cancer. Although several, potential, clinical predictive markers have been identified, none so far have proven reliable. Herein we evaluated changes in serum alanine aminotransferase (ALT), which is upregulated by the accumulation of activated CD8+T cells in the liver, as a potentially reliable predictive marker. We retrospectively analyzed 265 patients with advanced malignancies at three institutions between 2016 and 2019. The patients received IO drug therapy. We defined the ALT ratio (ALR) as the serum ALT value at baseline / the highest serum ALT during IO drug therapy, then determined whether the ALR correlated with the objective response rate or progression-free survival. The median follow-up was 3.1 months. We observed objective responses in 65 patients. The ALR ranged from 0.19 to 32.2 (median 1.5), and a significant ALR increase was observed in responders (*p* < 0.001). In receiver operating characteristic analysis, ALR = 1.55 had the highest sensitivity and specificity. The patients with ALR < 1.55 had a significantly poorer PFS than those with ALR ≥ 1.55. A high ALR was associated with a tumor response and good PFS in patients with advanced malignancies. The ALR based on activated cytotoxic T lymphocyte dynamics is therefore a reliable predictive marker.

## Introduction

Programmed death-1 (PD-1) is inducibly expressed by activated cytotoxic T lymphocytes (CTLs). PD-1 interacts with its ligand, B7-H1, to deliver an inhibitory signal to CTLs^1,2^. Cytotoxic T lymphocyte–associated antigen 4 (CTLA-4) is also inducibly expressed on CTLs and interacts with its ligand, CD80 or CD86, to deliver an inhibitory signal to CTLs^3,4^. Both molecules inhibit antitumor immunity. Anti-PD-1 (nivolumab or pembrolizumab) and anti-CTLA-4 (ipilimumab) antibody block the inhibitory signal, thereby activating the CTLs against the cancer^5–8^. Recently, anti-PD-1 and anti-CTLA-4 antibodies were approved for use against several cancers^9,10^. These antibodies are comprehensively referred to as immune-oncology (IO) drugs. Nivolumab was effective against non-small-cell lung cancer (NSCLC)^11,12^, melanoma^13^, renal cell carcinoma (RCC)^14^, urothelial carcinoma (UC)^15,16^, squamous cell carcinoma of the head and neck (SCCHN)^17^, gastric cancer (GC)^18^, malignant mesothelioma (MM)^19^, Hodgkin’s lymphoma^20^, and hepatocellular carcinoma^21^. Pembrolizumab, another anti-PD-1 antibody, was also effective against NSCLC^22^, melanoma^23^, RCC^24^, SCCHN^25^, UC^26^, and Merkel cell carcinoma^27^. Ipilimumab was effective against melanoma^10^ and RCC^28^.

Despite their efficacy, IO drugs benefit only a small number of patients. Therefore, reliable predictive markers allowing the identification of responders and non-responders are crucial to choosing the optimal treatment. Several studies have discussed predictive markers, such as PD-L1 expression^29^, overall mutational burden^30^, leukocyte count^31–33^, lactate dehydrogenase^33,34^, C-reactive protein^35^, and adverse events^36–38^, but as of yet, no reliable predictive marker has been found^39^.

CTLs, which are activated by IO drugs, accumulate in the liver and undergo apoptosis^40,41^. Dong et al. showed a greater accumulation of CTLs in the liver of B7-H1-deficient mice than in wild-type mice^42^. In the present study, we evaluated changes in the serum alanine aminotransferase (ALT) level, which is a marker of liver injury, as a mechanism-based predictive marker for IO drug therapy.

## Results

### Patient characteristics

In total, 265 patients (134 with NSCLC (92 with adenocarcinoma and 42 with SCC); 38 with GC; 52 with RCC; 35 with UC; three with MM; and three with an unknown primary cancer) were enrolled (Table 1). The median follow-up period was 3.1 months (0.2–46.3). The average age was 69.3 years (42–88), and the male: female ratio was 193 : 72. We observed objective responses in 65 patients (44 with NSCLC (26 with adenocarcinoma and 18 with SCC); four with GC, ten with RCC; five with UC; one with MM; and one with an unknown primary cancer).

**Table 1:** Patient characteristics (n = 265).

Age	Average	69.3 years
	Range	42–88 years
		Number of patients (%)
Sex	Male	193 (73)
	Female	72 (27)
I.O. Drugs	Nivolumab	180 (68)
	Pembrolizumab	71 (27)
	Nivolumab and Ipilimumab	14 (5)
Cancer type		
Non-small-cell lung cancer		134
Adenocarcinoma	Nivolumab	66 (25)
	Pembrolizumab	26 (10)
Squamous cell carcinoma	Nivolumab	33 (12)
	Pembrolizumab	9 (3)
Gastric cancer	Nivolumab	38 (14)
Renal cell carcinoma	Nivolumab	38 (14)
	Nivolumab and Ipilimumab	14 (5)
Urothelial carcinoma	Pembrolizumab	35 (13)
Malignant mesothelioma	Nivolumab	3 (2)
Unknown primary cancer	Nivolumab	2 (1.5)
	Pembrolizumab	1 (0.5)

### ALR according to the response to immunotherapy

**Figure 1:**
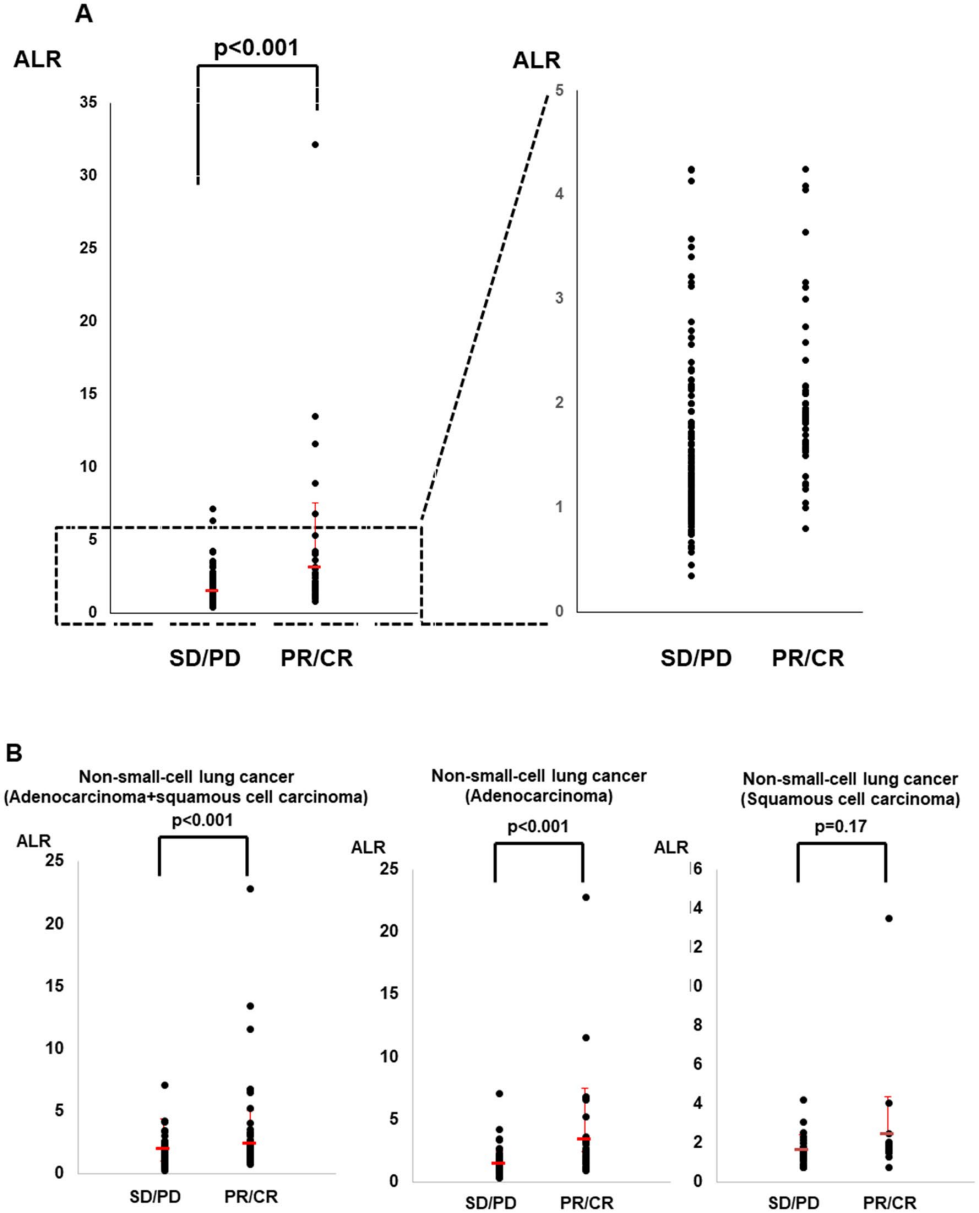

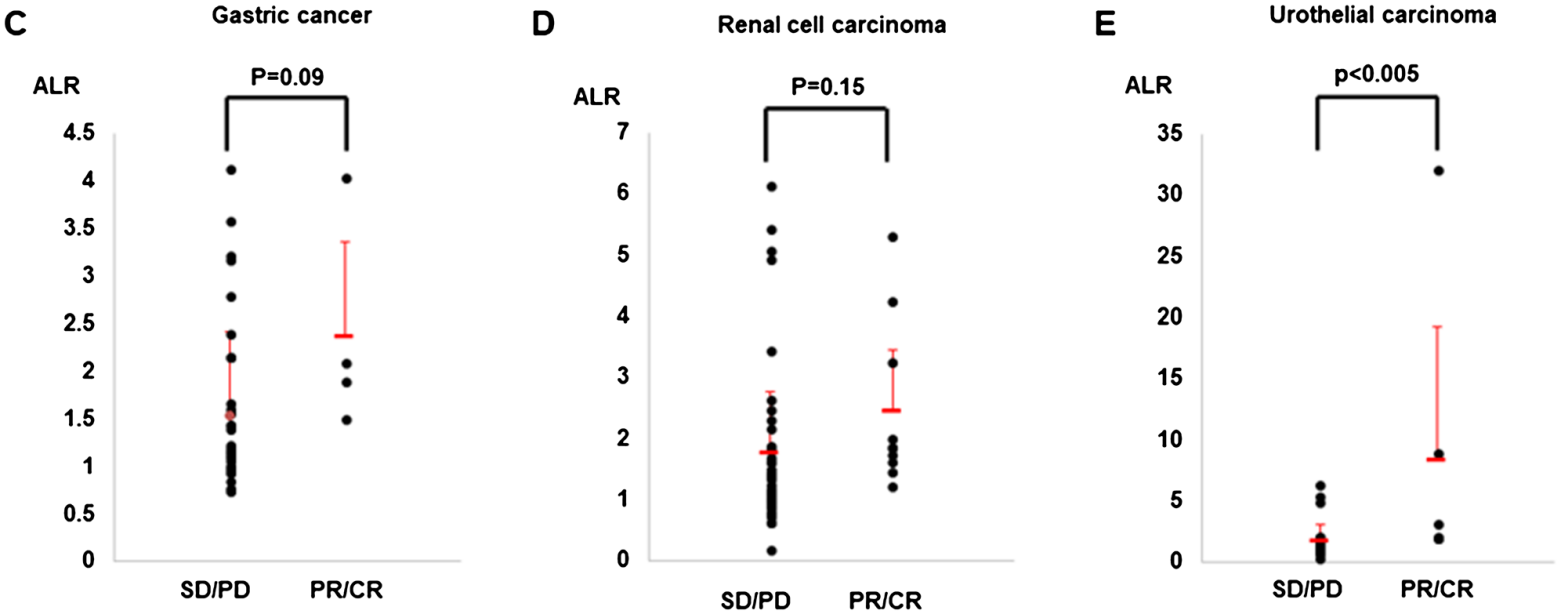
(**A**) ALR according to the response to immunotherapy (SD/PD, stable/progressive disease; PR/CR, partial/complete response). ALR according to the response to immunotherapy against lung cancer (**B**), gastric cancer (**C**), renal cell carcinoma (**D**), and urothelial carcinoma (**E**). Bars represent the mean ALR. Error bars show the mean standard deviation.

**Figure 2:**
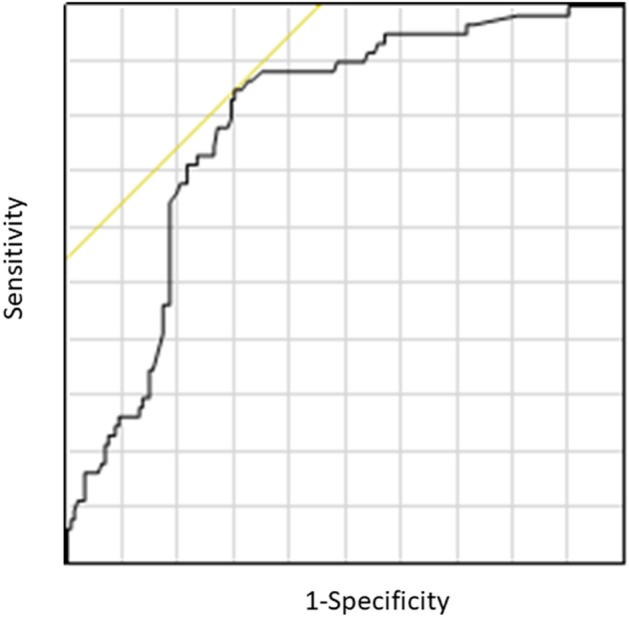
Receiver operating curve (ROC) analysis based on the ALR for the tumor response. In this model, the sensitivity was 85.0%, the specificity was 70.0%, and the AUC was 0.790. *p* < 0.001. AUC, area under the curve.

**Figure 3:**
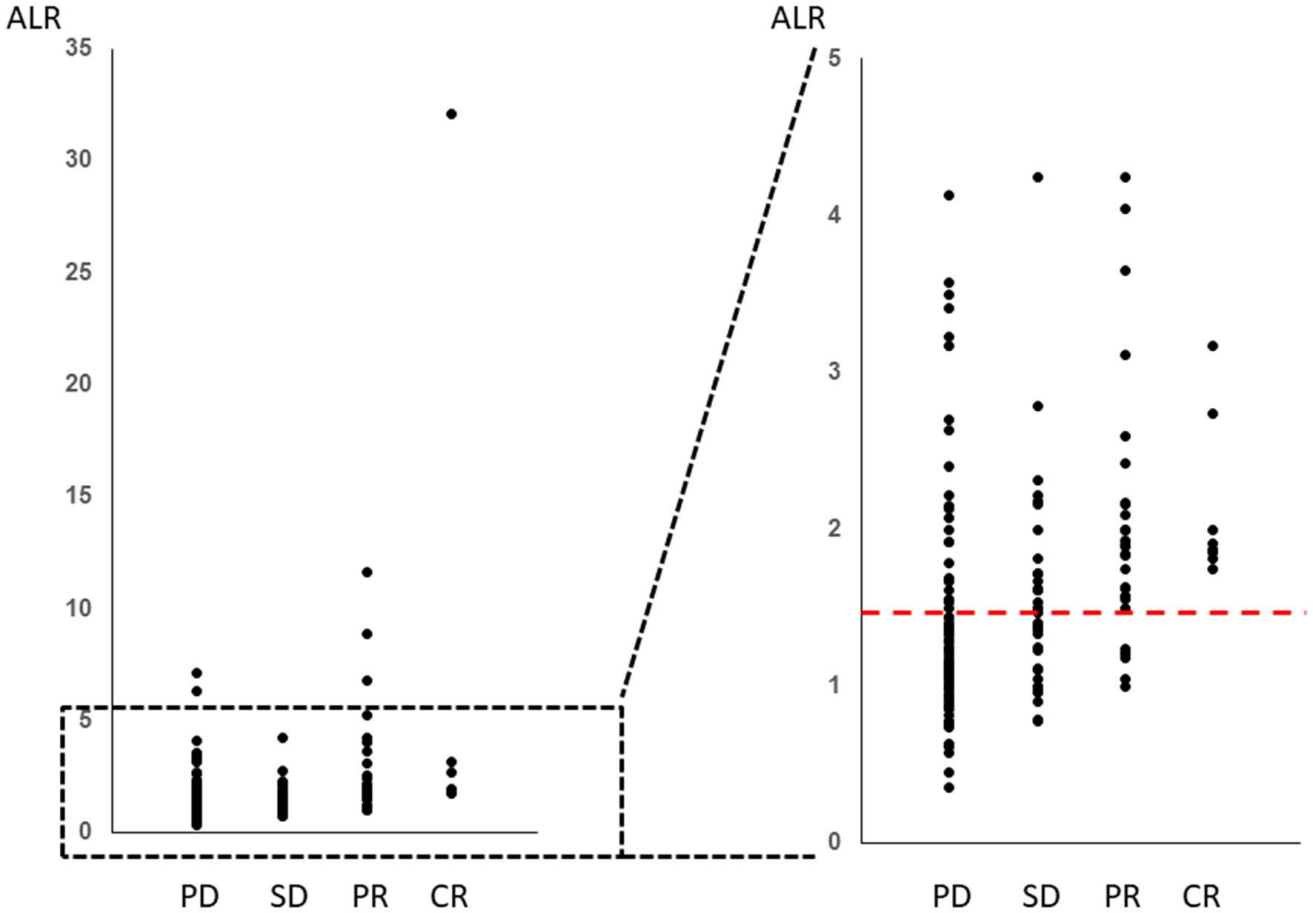
ALR according to the response to immunotherapy (*CR* complete response; *PR* partial response; *SD* stable disease; *PD* progressive disease).

The ALR ranged from 0.19 to 32.2 (median 1.5). We observed a significant increase in the ALR among responders (*p* < 0.001, Fig. 1). The ALR was able to predict the tumor response, as determined by ROC curve sensitivity (0.85) and specificity (0.70) (Fig. 2). We observed an increase in the ALR (≥ 1.55) in 52 of 65 responders (42 of 55 patients with a PR and in all ten patients with a CR) and 67 of 200 non-responders (27 of 55 patients with SD and 40 of 145 patients with PD) (odds ratio:9.7, *p* < 0.0001, Fig. 3). In the 67 non-responders with an elevated ALR, 12 experienced exacerbation or liver metastasis, nine experienced adverse events associated with immunotherapy (liver toxicity, etc.), and ten showed a mixed response, in which some tumors shrank, but PR was not achieved or a new metastasis occurred.

### Timing of the serum ALT increase in responders

**Figure 4:**
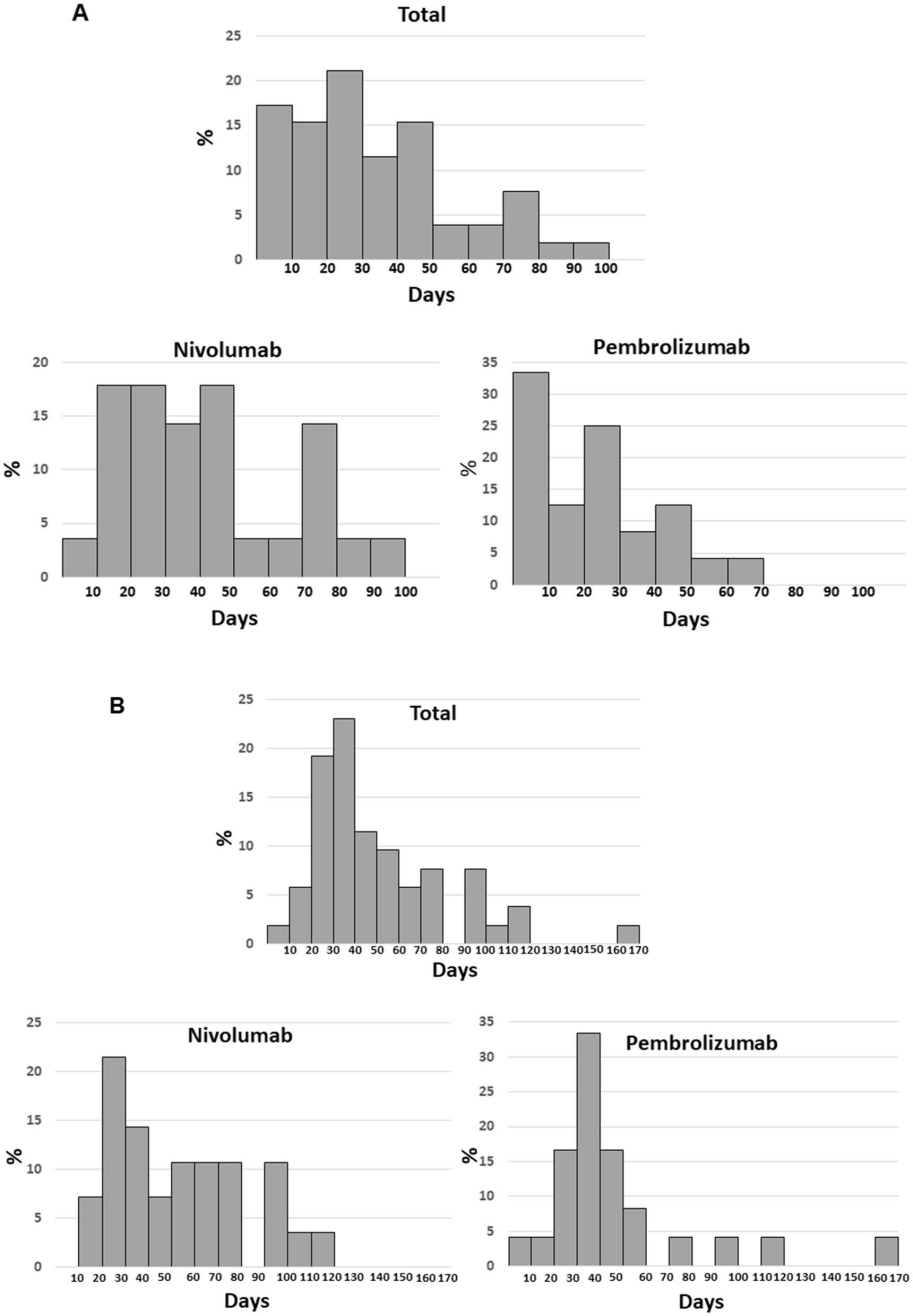
(**A**) Timing of the serum ALT increase in responders. (**B**) Variations in the timing of the response after the serum ALT increase in responders.

We analyzed the timing of the serum ALT increase in 52 responders (Fig. 4A). The serum ALT increased in 28 (54%) and 42 (81%) patients within 30 and 60 days, respectively, following the initial drug infusion. In all the responders, a serum ALT increase occurred within 3 months. Next, we evaluated the differences by drug. In patients receiving nivolumab, one (4%), 11 (40%), and 21 (75%) of 28 patients showed a serum ALT increase within ten, 30, and 60 days, respectively, following the initial drug infusion. In patients receiving pembrolizumab, eight (35%), 17 (71%), and 23 (100%) of 23 patients showed a serum ALT increase within ten, 30, and 60 days, respectively, following the initial drug infusion. Variations in the timing of the response after the serum ALT increase were also analyzed (Fig. 4B). In 14 (27%), 37 (71%), and 44 (85%) of 52 patients, the response developed within 30, 60, and 90 days, respectively, following the serum ALT increase. In patients receiving nivolumab, one (4%), 11 (40%), and 21 (75%) of 28 patients developed the response within ten, 30, and 60 days, respectively, following the initial drug infusion. In patients receiving pembrolizumab, eight (35%), 17 (71%), and 23 (100%) of 23 patients developed the response within ten, 30, and 60 days, respectively, following the initial drug infusion.

### Progression-free survival estimated using the ALR

**Figure 5:**
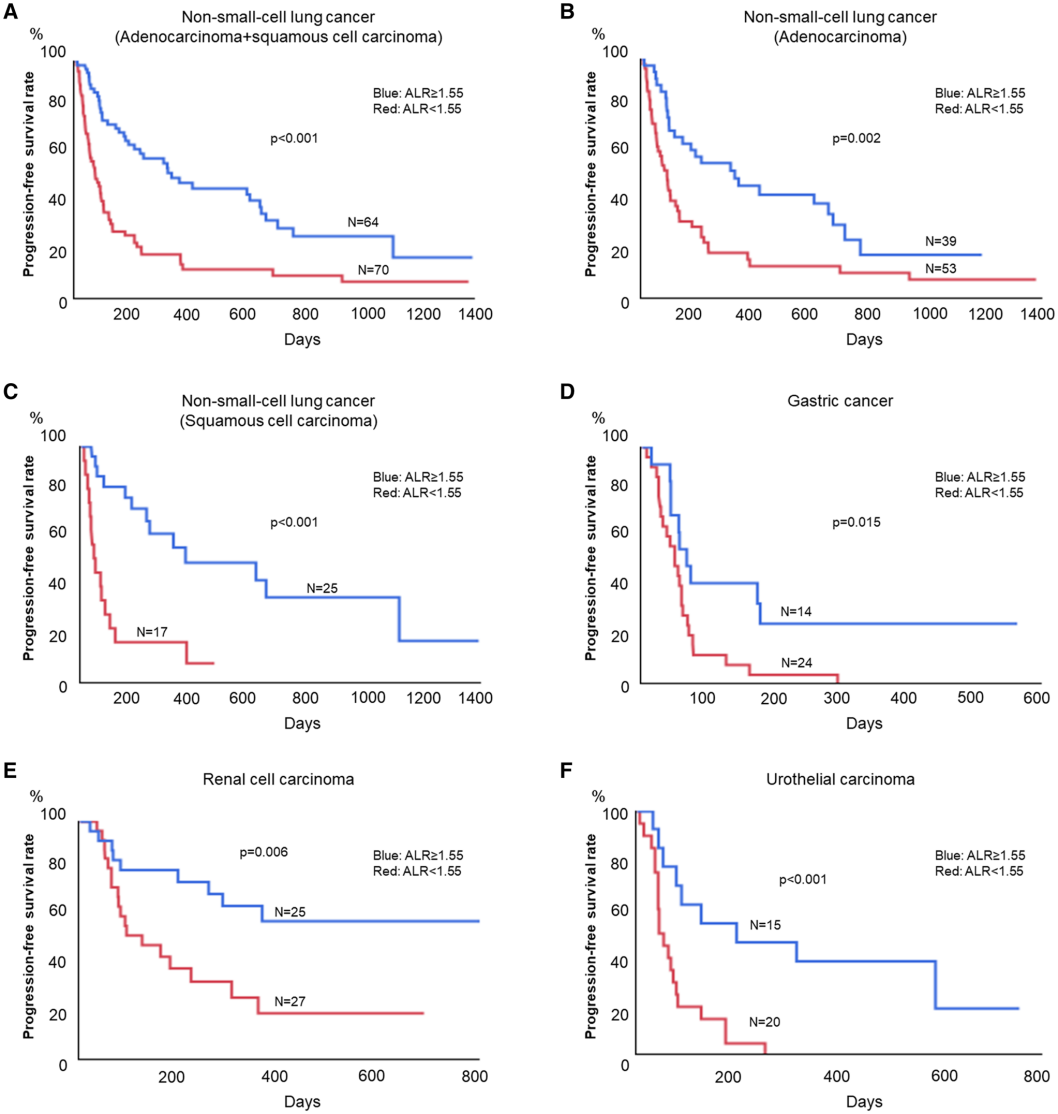
Progression-free survival (PFS) estimated using the ALR for non-small cell lung cancer (**A**), adenocarcinoma of the lung (**B**), squamous cell carcinoma of the lung (**C**), gastric cancer (**D**), renal cell carcinoma (**E**) and urothelial carcinoma (**F**).

Among patients with NSCLC, GC, RCC, and UC, an ALR < 1.55 was associated with significantly poorer PFS than ALR ≥ 1.55. The median PFS among patients with NSCLC with ALR < 1.55 and ALR ≥ 1.55 was 2.6 and 12 months, respectively (*p* < 0.001, Fig. 5A). Subgroup analysis performed for tumor histology (adenocarcinoma or squamous cell carcinoma) demonstrated that elevated ALR remained a significant prognostic factor. The median PFS in patients with adenocarcinoma with an ALR < 1.55 and ALR ≥ 1.55 was 2.9 and 11.2 months, respectively (*p* = 0.002, Fig. 5B). The median PFS in patients with squamous cell carcinoma with an ALR < 1.55 and ALR ≥ 1.55 was 1.5 and 20.3 months, respectively (*p* < 0.001, Fig. 5C). Elevated ALR was a significant prognostic factor in patients with gastric cancer, RCC, and UC. The median PFS in patients with gastric cancer with an ALR < 1.55 and ALR ≥ 1.55 was 1.8 and 2.2 months, respectively (*p* = 0.015, Fig. 5D). The median PFS in patients with RCC with an ALR < 1.55 and ALR ≥ 1.55 was 4.1 months and not achieved, respectively (*p* = 0.006, Fig. 5E). The median PFS in patients with UC with an ALR < 1.55 and ALR ≥ 1.55 was 1.4 and 6.5 months, respectively (*p* < 0.001, Fig. 5F).

## Discussion

Reliable predictive markers are needed to distinguish responders from non-responders. Although several candidate markers have already been identified, they have some limitations^29–38^. Our study showed that ALR may serve as a novel predictive marker based on the dynamics of activated CTLs, and its utility may be improved by further basic research.

Crispe et al. revealed that CTLs activated by an antigen were deleted in the liver, suggesting either a preferential accumulation in the liver of activated CTLs undergoing apoptosis (the graveyard hypothesis) or a trap for activated CTLs in the liver for subsequent killing (the killing field hypothesis)^40,41^. In B7-H1 deficient mice, activated PD-1+ CD8 T cells accumulated in the liver^42^. We surmised that the accumulation and deletion of activated CTLs in the liver might impose a burden on the liver, which could induce hepatopathy and elevate serum liver enzymes^43^. After assessing both serum aspartate aminotransferase and ALT, we found that serum ALT was a better predictive marker (Supplementary Fig. 1).

The timing of the serum ALT increase varies for each IO drug. In most patients receiving pembrolizumab or a combination of nivolumab and ipilimumab, the timing of serum ALT elevation was 1–3 weeks following the initial drug infusion. However, the timing for nivolumab was different, varying from soon after the initial infusion to later in some cases. This difference may depend on the affinity of each IO drug to the PD-1 molecule. An antibody with a strong affinity might affect the immune system sooner than an antibody with a weaker affinity; pembrolizumab has a stronger affinity than nivolumab to PD-1 expressing T cells^44^. Usually, serum ALT rises immediately prior to the finding of tumor shrinkage on a radiologic modality. These findings suggested that ALR might serve as a predictive marker of IO drug therapy.

The present study has the limitations inherent in any retrospective analysis. The patient population varied widely. The ALR was an effective predictive marker, but presented certain problems, such as the occurrence of false negative and false positive cases. We observed no increase in the ALR in 11 (17.0%) responders while we found an increase in 67 (33.5%) non-responders.

There are several possible explanations for the false negatives. First, our timing of ALT analysis was inadequate in that we failed to perform the analysis when the ALT had reached its highest value. Second, because injury to the liver was small, we did not observe elevations in the serum ALT levels. There are two reasons for the low level of injury to the liver. First, the shrunken tumor volume was too small to induce sufficient CTL activation to cause hepatopathy; in effect, there was insufficient injury to the liver to elevate the ALT level. Another reason for the minimal injury to the liver was exceptional liver function. There are also several possible explanations for the false positive results. In twelve patients, a new liver metastasis or the progression of an existing liver metastasis induced a serum ALT increase. In nine patients, other reasons, for example, autoimmune hepatitis, autoimmune cholangitis, septic shock, other drug-induced hepatopathy, etc., accounted for the elevated ALT. In ten patients, despite the attainment of SD or PD according to the RECIST criteria, a portion of the tumors showed shrinkage. We were able to identify other, clinical causes of elevated ALT as well; after excluding 21 cases of elevated ALT due to other causes from the cohort and adding ten cases showing some tumor shrinkage to the responder group, the odds ratio rose from 9.7 to 16.8. In clinical practice, the ALR may serve as a very promising predictor of the response to IO drug therapy when transitioning to the next treatment. These circumstances should be taken into consideration, and ALR should be used carefully.

The PFS duration was significantly longer in the high ALR group than in the low ALR group for four types of cancer, suggesting that the ALR can help differentiate between long and short SD among non-responders. The ALR is useful for patients receiving cancer immunotherapy because it facilitates judging whether immunotherapy should be continued or switched to an alternative treatment. Previous studies have reported several, reliable predictive markers for IO drug therapy, including PD-L1 expression and the tumor mutation burden^39^. However, these factors have limitations due to cost-related concerns; these markers require additional analysis, the involvement of specialists or the use of expensive equipment. Some studies revealed that in contrast to these markers, certain blood and clinical markers, such as the leukocyte count, lactate dehydrogenase, and adverse events, can be routinely used^39^. ALR is also one such available blood marker. However, in this respect the ALR, based on CTL dynamics, is an effective marker of immunotherapy and has the potential to serve as a simple, reliable prognostic marker in risk stratification and provide better treatment allocation in cancer patients.

A high ALR was associated with the tumor response and PFS in patients with various types of cancer. The accumulation and deletion of activated CTLs in the liver might impose a burden on the liver, which induce the elevation of ALR. Thus, an increase in ALT levels during IO drug therapy is reliable because the ALR is associated with CTL dynamics.

Further studies aimed at refining the use of the ALR as a predictive marker based on the mechanism of IO drug therapy are warranted.

## Materials and methods

### Patients

Between 2016 and 2019, we reviewed the medical records of 265 patients with advanced malignancies at Tokyo Metropolitan Tama Medical Center, Kanto Rosai Hospital, and Tomishiro-Chuo Hospital who received single-agent nivolumab, pembrolizumab or a combination nivolumab and ipilimumab treatment.

The present study was approved by the ethical review board of Tama Medical Center, Kanto Rosai Hospital, and Tomishiro Central Hospital and was conducted in accordance with the principles of the Declaration of Helsinki and Good Clinical Practice Guidelines. Comprehensive iInformed consent was obtained from all the participants.

### Treatment and response assessment

Patients received nivolumab 3 mg/kg every 2 weeks, pembrolizumab 200 mg every 3 weeks or nivolumab 240 mg every 3 weeks plus ipilimumab 3 mg per kilogram every 2 weeks in four doses, followed by nivolumab 240 mg every 2 weeks.

All the patients underwent blood testing from before the commencement of IO drug therapy to the end of follow-up at intervals of 1–2 weeks. We defined ALR as the serum ALT value at baseline / the highest serum ALT during IO drug therapy.

Computed tomography or magnetic resonance imaging was performed at baseline and repeated at 8- to 12-week intervals. The clinical tumor response during treatment was assessed using the Response Evaluation Criteria in Solid Tumors (RECIST) 1.1 based on the patients’ medical records. We defined a clinical benefit as a complete response (CR), partial response (PR) or stable disease (SD). We defined progression-free survival (PFS) as the time from the commencement of immunotherapy to the date of progression.

### Statistics

Statistical analyses were performed using the JMP® software package, with *p* < 0.05 indicating statistical significance. We compared continuous variables using the two-tailed unpaired Student’s t-test. To predict the response to, and prognosis of, IO drug therapy, we determined the optimal cutoff value of the ALR based on the receiver operating characteristic (ROC) curve and the area under the ROC curve (AUC). We constructed the distribution of the recurrence-free survival (RFS) rate using the Kaplan–Meier method.

## Supplementary Information

Supplementary Figure S1.

## References

[1] Dong H, Zhu G, Tamada K, Chen L (1999). B7–H1, a third member of the B7 family, co-stimulates T-cell proliferation and interleukin-10 secretion. Nat. Med..

[2] Ishida Y, Agata Y, Shibahara K, Honjo T (1992). Induced expression of PD-1, a novel member of the immunoglobulin gene superfamily, upon programmed cell death. EMBO J..

[3] Salomon B, Bluestone JA (2001). Complexities of CD28/B7: CTLA-4 costimulatory pathways in autoimmunity and transplantation. Annu. Rev. Immunol..

[4] Melero I, Hervas-Stubbs S, Glennie M, Pardoll DM, Chen L (2007). Immunostimulatory monoclonal antibodies for cancer therapy. Nat. Rev. Cancer.

[5] Hirano F (2005). Blockade of B7–H1 and PD-1 by monoclonal antibodies potentiates cancer therapeutic immunity. Cancer Res..

[6] Azuma T (2008). B7–H1 is a ubiquitous antiapoptotic receptor on cancer cells. Blood.

[7] Chen L (2018). B7–H1 maintains the polyclonal T cell response by protecting dendritic cells from cytotoxic T lymphocyte destruction. Proc. Natl. Acad. Sci. U.S.A..

[8] O'Day SJ, Hamid O, Urba WJ (2007). Targeting cytotoxic T-lymphocyte antigen-4 (CTLA-4): a novel strategy for the treatment of melanoma and other malignancies. Cancer.

[9] Topalian SL (2012). Safety, activity, and immune correlates of anti-PD-1 antibody in cancer. N. Engl. J. Med..

[10] Hodi FS (2010). Improved survival with ipilimumab in patients with metastatic melanoma. N. Engl. J. Med..

[11] Borghaei H (2015). Nivolumab versus docetaxel in advanced nonsquamous non-small-cell lung cancer. N. Engl. J. Med..

[12] Brahmer J (2015). Nivolumab versus docetaxel in advanced squamous-cell non-small-cell lung cancer. N. Engl. J. Med..

[13] Weber JS (2015). Nivolumab versus chemotherapy in patients with advanced melanoma who progressed after anti-CTLA-4 treatment (CheckMate 037): a randomised, controlled, open-label, phase 3 trial. Lancet. Oncol..

[14] Motzer RJ (2015). Nivolumab versus Everolimus in Advanced Renal-Cell Carcinoma. N. Engl. J. Med..

[15] Sharma P (2016). Nivolumab monotherapy in recurrent metastatic urothelial carcinoma (CheckMate 032): a multicentre, open-label, two-stage, multi-arm, phase 1/2 trial. Lancet Oncol..

[16] Sharma P (2017). Nivolumab in metastatic urothelial carcinoma after platinum therapy (CheckMate 275): a multicentre, single-arm, phase 2 trial. Lancet Oncol..

[17] Ferris RL (2016). Nivolumab for recurrent squamous-cell carcinoma of the head and neck. N. Engl. J. Med..

[18] Kang YK (2017). Nivolumab in patients with advanced gastric or gastro-oesophageal junction cancer refractory to, or intolerant of, at least two previous chemotherapy regimens (ONO-4538-12, ATTRACTION-2): a randomised, double-blind, placebo-controlled, phase 3 trial. Lancet.

[19] Scherpereel A (2019). Nivolumab or nivolumab plus ipilimumab in patients with relapsed malignant pleural mesothelioma (IFCT-1501 MAPS2): a multicentre, open-label, randomised, non-comparative, phase 2 trial. Lancet Oncol..

[20] Ansell SM (2015). PD-1 blockade with nivolumab in relapsed or refractory Hodgkin's lymphoma. N. Engl. J. Med..

[21] El-Khoueiry AB (2017). Nivolumab in patients with advanced hepatocellular carcinoma (CheckMate 040): an open-label, non-comparative, phase 1/2 dose escalation and expansion trial. Lancet.

[22] Reck M (2016). Pembrolizumab versus chemotherapy for PD-L1-positive non-small-cell lung cancer. N. Engl. J. Med..

[23] Robert C (2015). Pembrolizumab versus Ipilimumab in Advanced Melanoma. N. Engl. J. Med..

[24] Rini BI (2019). Pembrolizumab plus axitinib versus sunitinib for advanced renal-cell carcinoma. N. Engl. J. Med..

[25] Bauml J (2017). Pembrolizumab for platinum- and cetuximab-refractory head and neck cancer: results from a single-arm, phase II study. J. Clin. Oncol..

[26] Bellmunt J (2017). Pembrolizumab as second-line therapy for advanced urothelial carcinoma. N. Engl. J. Med..

[27] Nghiem PT (2016). PD-1 blockade with pembrolizumab in advanced merkel-cell carcinoma. N. Engl. J. Med..

[28] Motzer RJ (2018). Nivolumab plus Ipilimumab versus sunitinib in advanced renal-cell carcinoma. N. Engl. J. Med..

[29] Davis AA, Patel VG (2019). The role of PD-L1 expression as a predictive biomarker: an analysis of all US Food and Drug Administration (FDA) approvals of immune checkpoint inhibitors. J. Immunother. Cancer.

[30] Le DT (2015). PD-1 blockade in tumors with mismatch-repair deficiency. N. Engl. J. Med..

[31] Gebhardt C (2015). Myeloid cells and related chronic inflammatory factors as novel predictive markers in melanoma treatment with ipilimumab. Clin. Cancer Res..

[32] Martens A (2016). Baseline peripheral blood biomarkers associated with clinical outcome of advanced melanoma patients treated with ipilimumab. Clin. Cancer Res..

[33] Nakamura Y (2016). Nivolumab for advanced melanoma: pretreatment prognostic factors and early outcome markers during therapy. Oncotarget.

[34] Diem S (2016). Serum lactate dehydrogenase as an early marker for outcome in patients treated with anti-PD-1 therapy in metastatic melanoma. Br. J. Cancer.

[35] Simeone E (2014). Immunological and biological changes during ipilimumab treatment and their potential correlation with clinical response and survival in patients with advanced melanoma. Cancer Immunol. Immunother..

[36] Hua C (2016). Association of vitiligo with tumor response in patients with metastatic melanoma treated with pembrolizumab. JAMA Dermatol..

[37] Rogado J (2019). Immune-related adverse events predict the therapeutic efficacy of anti-PD-1 antibodies in cancer patients. Eur. J. Cancer.

[38] Weber JS (2017). Safety profile of nivolumab monotherapy: a pooled analysis of patients with advanced melanoma. J. Clin. Oncol..

[39] Hopkins AM (2017). Predicting response and toxicity to immune checkpoint inhibitors using routinely available blood and clinical markers. Br. J. Cancer.

[40] Crispe IN (2003). Hepatic T cells and liver tolerance. Nat. Rev. Immunol..

[41] Crispe IN, Dao T, Klugewitz K, Mehal WZ, Metz DP (2000). The liver as a site of T-cell apoptosis: graveyard, or killing field?. Immunol. Rev..

[42] Dong H (2004). B7–H1 determines accumulation and deletion of intrahepatic CD8(+) T lymphocytes. Immunity.

[43] Gowda S (2009). A review on laboratory liver function tests. Pan Afr. Med. J..

[44] Tang S, Kim PS (2019). A high-affinity human PD-1/PD-L2 complex informs avenues for small-molecule immune checkpoint drug discovery. Proc. Natl. Acad. Sci. U.S.A..

