# Supplementary figures and images for: Serum alanine aminotransferase as an early marker of outcomes in patients receiving anti-PD-1 or anti-CTLA-4 antibody
Source: Sci Rep. 2021 May 13;11:10264. doi: 10.1038/s41598-021-88744-0 (PMC8119734; doi:10.1038/s41598-021-88744-0)

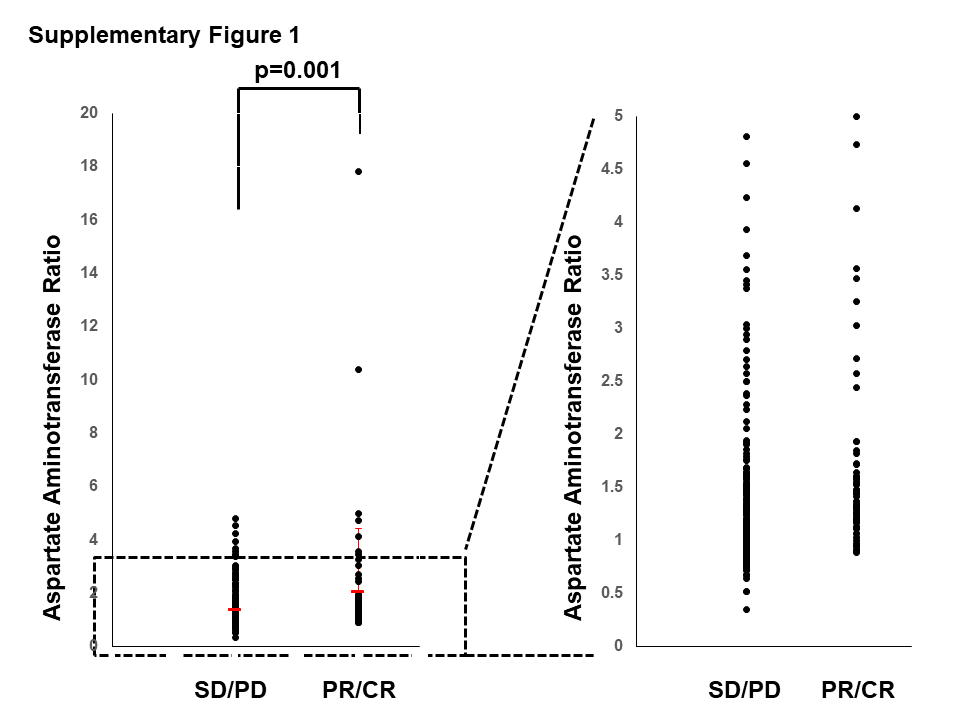

Supplement: Supplementary file 1 — Supplementary Figure S1. [file 41598_2021_88744_MOESM1_ESM.tif]
